# Reduced toxicity in the treatment of locally advanced rectal cancer: a comparison of volumetric modulated arc therapy and 3D conformal radiotherapy

**DOI:** 10.1186/s12885-015-1812-x

**Published:** 2015-10-20

**Authors:** Leif Hendrik Dröge, Hanne Elisabeth Weber, Manuel Guhlich, Martin Leu, Lena-Christin Conradi, Jochen Gaedcke, Steffen Hennies, Markus Karl Herrmann, Margret Rave-Fränk, Hendrik Andreas Wolff

**Affiliations:** 1 Department of Radiotherapy and Radiation Oncology, University Medical Center Göttingen, Robert-Koch-Straße 40, 37075 Göttingen, Germany; 2 Department of General, Visceral and Pediatric Surgery, University Medical Center Göttingen, Göttingen, Germany; 3 Present address: Radiologie München, Burgstrasse 7, 80331 München, Germany; 4 MVZ Klinik Dr. Hancken, Strahlentherapie und Radioonkologie, Stade, Germany

**Keywords:** Rectal cancer, Chemoradiotherapy, 3D conformal radiotherapy, Volumetric modulated arc therapy, Tumor regression grading, Acute toxicity, Late toxicity

## Abstract

**Background:**

Excellent dosimetric characteristics were demonstrated for volumetric modulated arc therapy (VMAT) in preoperative chemoradiotherapy (CRT) for locally advanced rectal cancer (LARC). In a single-center retrospective analysis, we tested whether these advantages may translate into significant clinical benefits. We compared VMAT to conventional 3D conformal radiotherapy (3DCRT) in patients, homogeneously treated according to the control arm of the CAO/ARO/AIO-04 trial.

**Methods:**

CRT consisted of pelvic irradiation with 50.4/1.8Gy by VMAT (*n* = 81) or 3DCRT (*n* = 107) and two cycles of 5-fluorouracil. Standardized total mesorectal excision surgery was performed within 4–6 weeks. The tumor regression grading (TRG) was assessed by the Dworak score. Acute and late toxicity were evaluated via the Common Terminology Criteria for Adverse Events and the Late effects of normal tissues scale, respectively. Side effects greater than or equal to grade 3 were considered high-grade.

**Results:**

Median follow-up was 18.3 months in the VMAT group and 61.5 months in the 3DCRT group with no differences in TRG between them (*p* = 0.1727). VMAT treatment substantially reduced high-grade acute and late toxicity, with 5 % versus 20 % (*p* = 0.0081) and 6 % vs. 22 % (*p* = 0.0039), respectively. With regard to specific organs, differences were found in skin reaction (*p* = 0.019) and proctitis (*p* = 0.0153).

**Conclusions:**

VMAT treatment in preoperative CRT for LARC showed the potential to substantially reduce high-grade acute and late toxicity. Importantly, we could demonstrate that VMAT irradiation did not impair short-term oncological results. We conclude, that the reduced toxicity after VMAT irradiation may pave the way for more efficient systemic therapies, and hopefully improved patient survival in the multimodal treatment of LARC.

**Electronic supplementary material:**

The online version of this article (doi:10.1186/s12885-015-1812-x) contains supplementary material, which is available to authorized users.

## Background

Preoperative chemoradiotherapy (CRT) for locally advanced rectal cancer (LARC), followed by standardized total mesorectal excision (TME) surgery, results in excellent local control rates, but distant failure compromises patients’ survival [1, 2]. To reduce distant failure risk, clinical trials aim to intensify systemic treatment, at the hazard of increased toxicity and quality of life impairment [3–5]. Such strategy requires the optimization of any local therapy, including radiotherapy (RT), in terms of efficacy and tolerability.

Advanced RT techniques, namely intensity-modulated radiotherapy (IMRT), volumetric modulated arc therapy (VMAT), and proton therapy showed excellent target volume coverage and organs at risk sparing in dosimetric studies [6–8]. To a very limited extent, clinical studies on LARC irradiation reported enhanced tumor response [9] and reduced acute toxicity [9, 10] when IMRT was compared to conventional 3D conformal radiotherapy (3DCRT). A large-scale direct comparison of clinical results after VMAT and 3DCRT has not been reported to date.

Based on promising dosimetric results, VMAT was introduced to our clinic and gradually replaced 3DCRT for LARC since 2009. The purpose of the present single-center study was to compare VMAT-treated patients with 3DCRT-treated patients in terms of tumor response, acute and late toxicity.

## Methods

### Patients

The database at our institution contained 188 patients who were consecutively treated with neoadjuvant CRT and concurrent 5-fluorouracil for non-metastatic LARC from 2005 to 2014. The diagnosis was assured via rigid endoscopy with histologic sampling. The clinical tumor stage was assessed by endoscopic ultrasound and pelvic MRI scan.

All patients were treated according to the control arm of the CAO/ARO/AIO-04 trial [EudraCT no.: 2006-002385-20]. This multicenter, randomized phase III trial investigated the addition of oxaliplatin to multimodal treatment of LARC. Patients were assigned to receive either standard neoadjuvant 5-fluorouracil-based CRT, TME surgery, and adjuvant 5-fluorouracil chemotherapy (control arm), or neoadjuvant CRT with 5-fluorouracil/oxaliplatin, TME surgery, and adjuvant 5-fluorouracil/oxaliplatin/leucovorin (investigational arm) [4]. At our institution, all the LARC patients were highly homogeneously treated by a specialized interdisciplinary group in the context of the Clinical Research Unit 179, funded by the German Research Foundation (DFG). The investigations were conducted according to Declaration of Helsinki principles. The Ethics Committee at the University of Göttingen approved the study, and patients gave informed consent in written form.

### Chemoradiotherapy

RT was applied with linear accelerator photons to a reference dose of 50.4Gy in 1.8Gy fractions. Patients were positioned in abdominal position on a belly board. The clinical target volume (CTV) and the organs at risk were outlined on the basis of the planning CT scan and the diagnostic MRI scan, using the Eclipse system (v8.9, Varian Medical Systems). The CTV included the primary tumor and the mesorectal, presacral and internal iliac lymph nodes [4]. The planning target volume (PTV) was defined by enlarging the CTV in all directions by 10 mm. Patients were treated according to respective technical standards. Conventional 3DCRT was used from 2005 to 2012, while VMAT superseded 3DCRT as of 2009.

The treatment plans were calculated according to ICRU recommendations. The dose was defined at the ICRU 50 reference point. The isodose curve representing 95 % of the prescribed dose had to encompass the entire PTV and the maximum dose to the PTV was limited to <107 % of the prescribed dose [11, 12]. The aim was to minimize the dose to the organs at risk, using these constraints [7]: bladder ≥40Gy in ≤50 % volume; small bowel ≥50Gy in ≤10 cm^3^ volume and ≥40Gy in ≤100 cm^3^ volume, whereas individual loops of small bowel were contoured.

As described before, the 3DCRT was applied using a three-field technique. The beam angles were 0, 90 and 270°. The photon energies were 6 MeV (beam direction, 0°) and 20 MeV (beam directions, 90 and 270°). A multi-leaf collimator (Millennium 120, Varian Medical Systems) was used to shape the fields. Wedges (45° or 60°) were used in lateral fields to obtain homogeneous dose distribution. VMAT was carried out using RapidArc© (Varian Medical Systems) with two full arcs, and with a photon energy of 6 MeV. A single arc was arranged into 177 control points (1 control point about every 2° of gantry) [7].

The concurrent chemotherapy for all patients consisted of 5-fluorouracil (1000 mg/m^2^ on days 1–5 and 29–33 of the RT). Standardized TME surgery was performed within 4–6 weeks, followed by 4 cycles of bolus 5-fluorouracil (500 mg/m^2^).

### Tumor response/ toxicity assessment

The tumor staging in the resected specimen was based on the sixth edition of the TNM classification [13]. The tumor regression grading (TRG) was assessed by the quantification of the ratio of tumor tissue versus fibrotic tissue (Dworak score) [14].

Acute toxicity was assessed via the National Cancer Institute Common Terminology Criteria for Adverse Events, version 3.0 [15]. A minimum of weekly examinations by the treating radiation oncologist and weekly blood samples were mandatory. After CRT, patients were closely monitored for at least 2 weeks and beyond that in the case of persisting acute toxicity. Late toxicity was evaluated according to the Late effects of normal tissues scale [16]. Patients were monitored for late toxicity at 3 months, and thereafter annually for up to 5 years. Toxicity of ≥ grade 3 was defined as high-grade toxicity.

### Statistical analysis

For the comparison of the patient characteristics, toxicity, surgery and histopathological parameters, the median and range are given for the continuous parameters while frequency and percentage are given for the categorical variables. The Chi-Square test and the Kruskal-Wallis test were used for comparison of categorical and continuous variables. The Kaplan-Meier method was used to compare the actuarial occurrence of late toxicity. *P*-values <0.05 were considered statistically significant. The analyses were performed using STATISTICA (v10.0.1011.0, StatSoft. Inc.).

## Results

### Patients

We included 188 patients who were treated from 05/2005 to 01/2014. Patient characteristics are presented in Table 1. The median patient age was 66 years (range, 35–86 years) with 64 years (range, 35–83 years) in the 3DCRT group, and 70 years (range, 43–86 years) in the VMAT group. The 3DCRT technique was used in 107 (56.9 %) patients and the VMAT technique in 81 (43.1 %).

**Table 1:** Patient characteristics

Characteristic	3D conformal radiotherapy		Volumetric modulated arc therapy		*p*
	No.	%	No.	%	
Gender					
female	30	28	23	28	0.9570
male	77	72	58	72	
Age, years					
≥70 years	38	36	41	51	*0.0378*
<70 years	69	64	40	49	
Body mass index [kg/m^2^]					
<20	2	2	0	0	*0.0453*
20–24.9	32	30	24	30	
25–26.9	15	14	19	24	
27–29.9	27	25	27	33	
≥30	31	29	11	13	
Clinical T category					
2	2	2	2	3	0.7843
3	96	90	70	86	
4	9	8	9	11	
Clinical N category					
negative	29	27	18	22	0.4441
positive	78	73	63	78	
Grading					
X	9	8	14	17	0.0806
1	1	1	0	0	
2	75	70	60	74	
3	21	20	7	9	
4	1	1	0	0	
Distance from anal verge					
0 to <6 cm	40	37	35	43	0.7212
6 to <12 cm	64	60	44	54	
12 to 16 cm	3	3	2	3	

The Chi-Square test and the Krusal-Wallis test were used for group comparisons

There were no differences in clinical T category, clinical N category, tumor grading and tumor distance from the anal verge. The VMAT group had a significantly larger proportion of patients with ≥70 years (*p* = 0.0378) and with a lower body mass index (*p* = 0.0453).

### Surgery and histopathological parameters

Surgery and histopathological data are presented in Table 2. The surgical procedures consisted of 123 low anterior resections (65 %) and 65 abdominoperineal resections (35 %). The frequency of low anterior resections and abdominoperineal resections was 68 (64 %) and 39 (36 %) in the 3DCRT group and 55 (68 %) and 26 (32 %) in the VMAT group, respectively. In tumors located within 0 to <6 cm from the anal verge, sphincter-saving surgery was performed in 9/40 patients (23 %) of the 3DCRT group and in 11/35 patients (31 %) of the VMAT group. A complete resection (R0) was achieved in 183 patients (97 %) with 104 (97 %) in the 3DCRT group and 79 (98 %) in the VMAT group. There were no differences regarding TRG, ypT category and ypN category.

**Table 2:** Surgery and histopathologic parameters

Characteristic	3D conformal radiotherapy		Volumetric modulated arc therapy		*p*
	No.	%	No.	%	
OP-method					
Low anterior resection	68	64	55	68	0.5356
Abdominoperineal resection	39	36	26	32	
Distance from anal verge 0 to <6 cm, sphincter-saving surgery					
Yes	9	23	11	31	0.3830
No	31	77	24	69	
R-status					
0	104	97	79	98	0.5330
1	1	1	2	2	
2	1	1	0	0	
X	1	1	0	0	
ypT-stage					
0	14	13	16	20	0.1397
1	11	10	5	6	
2	34	32	25	31	
3	42	39	35	43	
4	6	6	0	0	
ypN-stage					
0	69	65	57	70	0.5492
1	27	25	15	19	
2	11	10	9	11	
Tumor regression grading					
0 no regression	0	0	1	1	0.1727
1 minor regression	12	11	9	11	
2 moderate regression	34	32	32	40	
3 good regression	47	44	23	28	
4 total regression	14	13	16	20	

The Chi-Square test and the Krusal-Wallis test were used for group comparisons

Abbreviations: *R-status* resection status, *ypT* tumor stage after preoperative radiochemotherapy, *ypN* nodal stage after preoperative radiochemotherapy

### Acute toxicity

Acute organ toxicity data are presented in Table 3. Any kind of high-grade acute organ toxicity occurred in 25 of 188 patients (13 %), and was more frequent in the 3DCRT group with 21 of 107 patients (20 %) than in the VMAT group with four of 81 patients (5 %) (*p* = 0.0081). The 3DCRT patients had a significantly higher proportion of ≥ grade 3 skin reaction with 7 (7 %) in the 3DCRT group and 0 (0 %) in the VMAT group (*p* = 0.019). The frequency of ≥ grade 3 proctitis was higher in the 3DCRT cohort with 13 (12 %) for 3DCRT patients and 2 (2 %) for VMAT patients (*p* = 0.0153). In multi-group comparison, any kind of acute organ toxicity (*p* = 0.0113) and the skin reaction (*p* = 0.0056) were significantly more frequent in the 3DCRT group.

**Table 3:** Acute organ toxicity

Toxicity grade	3D conformal radiotherapy		Volumetric modulated arc therapy			
	No.	%	No.	%	Chi-square, *p*	Kruskal-Wallis, *p*
Skin reaction						
≥3	7	7	0	0	*0.0190*	
0	21	20	26	32		*0.0056*
1	42	39	39	48		
2	37	34	16	20		
3	7	7	0	0		
Proctitis						
≥3	13	12	2	2	*0.0153*	
0	15	14	9	11		0.0670
1	36	34	36	45		
2	43	40	34	42		
3	13	12	2	2		
Enteritis						
≥3	4	4	1	1	0.2907	
0	53	49	36	44		0.5898
1	34	32	29	36		
2	16	15	15	19		
3	4	4	1	1		
Cystitis						
≥3	3	3	1	1	0.4603	
0	63	59	39	48		0.2219
1	39	36	35	43		
2	2	2	6	7		
3	2	2	1	1		
4	1	1	0	0		
Balanitis						
0	106	99	81	100	^b^	
3	1	1	0	0		
Any kind of acute organ toxicity^a^						
≥3	21	20	4	5	*0.0081*	
0	4	4	2	3		*0.0113*
1	27	25	36	44		
2	55	51	39	48		
3	20	19	4	5		
4	1	1	0	0		

^a^The highest score of any acute organ toxicity per patient

^b^No statistical comparisons due to small groups of patients

Any kind of hematotoxicity (anemia, leucopenia, thrombopenia) ≥ grade 3 occurred in six patients (3 %) with four patients (4 %) in the 3DCRT group and two patients (3 %) in the VMAT group. The 3DCRT and VMAT group comparison showed no differences regarding hematotoxicity (Additional file 1: Table S1).

### Late toxicity

**Fig. 1:**
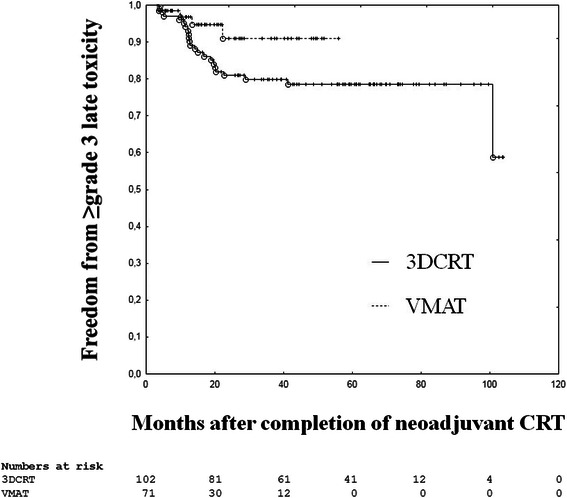
Freedom from ≥ grade 3 late toxicity. The Kaplan-Meier method was used to compare the occurrence of late toxicity in patients who underwent neoadjuvant chemoradiotherapy (CRT) with 3D conformal radiotherapy (3DCRT) or volumetric modulated arc therapy (VMAT)

Late toxicity data are presented in Table 4. Follow-up data were available for 173 patients (92 %) with 102 patients (95 %) in the 3DCRT group and 71 patients (88 %) in the VMAT group. The median follow-up time was 61.5 months (range, 4.0–105.7 months) in the 3DCRT group and 18.3 months (range, 4.0-59.2 months) in the VMAT group. The 2-year rates of freedom from high-grade late organ toxicity were 81 % for the 3DCRT group and 91 % for the VMAT group (Fig. 1). There were no differences regarding skin toxicity, proctitis and cystitis. Additional high-grade toxicity, namely enteritis (*n* = 4), urethral stricture (*n* = 1) or ureteral stenosis (*n* = 5) occurred in 10 3DCRT patients (10 %), while none of the VMAT patients (0 %) experienced these complications. Any kind of high-grade late organ toxicity occurred in 26 patients (15 %) with 22 patients (22 %) in the 3DCRT group and four patients (6 %) in the VMAT group (*p* = 0.0039). In multi-group comparison, high-grade late organ toxicity was significantly more frequent in the 3DCRT group (*p* = 0.0073).

**Table 4:** Late toxicity

Toxicity grade	3D conformal radiotherapy		Volumetric modulated arc therapy			
	No.	%	No.	%	Chi-square, *p*	Kruskal-Wallis, *p*
Skin						
≥3	2	2	0	0	0.2353	
0	83	81	64	90		0.3161
1	16	16	7	10		
2	1	1	0	0		
3	2	2	0	0		
Proctitis						
≥3	8	8	2	3	0.1635	
0	69	68	52	73		0.2498
1	11	11	10	14		
2	14	14	7	10		
3	6	6	0	0		
4	2	2	2	3		
Cystitis						
≥3	10	10	2	3	0.0752	
0	82	80	65	91		0.2271
1	8	8	2	3		
2	2	2	2	3		
3	9	9	2	3		
4	1	1	0	0		
Enteritis						
0	96	94	71	100	^b^	
1	2	2	0	0		
2	3	3	0	0		
3	1	1	0	0		
Lymphedema						
0	101	99	71	100	^b^	
1	1	1	0	0		
Urethral stricture						
0	101	99	71	100	^b^	
3	1	1	0	0		
Ureteral stenosis						
0	97	95	71	100	^b^	
3	5	5	0	0		
Any kind of the organ toxicity^a^						
≥3	22	22	4	6	*0.0039*	
0	44	43	47	66		*0.0073*
1	25	25	12	17		
2	11	11	8	11		
3	19	19	2	3		
4	3	3	2	3		

^a^The highest score of any late organ toxicity per patient

^b^No statistical comparisons due to small groups of patients

## Discussion

The use of IMRT and VMAT in the preoperative CRT of LARC is still rare, but dosimetric studies showed excellent target volume coverage and organs at risk sparing [7, 8, 10, 17]. Our group already demonstrated the superiority of VMAT over 3DCRT in a planning study with 25 patients, using the treatment protocol of the current study [7]. We analysed, whether these dosimetric advantages translate into significant clinical benefits. When directly comparing VMAT-treated patients with 3DCRT-treated patients in terms of tumor response, acute and late toxicity we found that VMAT provides equal tumor regression combined with reduced acute and late organ toxicity.

To assess tumor response, we used the standardized five-point TRG [14], and found no differences between VMAT-treated patients and 3DCRT-treated patients. Fokas et al. identified TRG as an independent prognostic factor for metastasis-free and disease-free survival of LARC patients treated according to the protocols of the CAO/ARO/AIO rectal cancer trials [18]. Thus TRG may reflect CRT effectiveness and our data could indicate that VMAT-treated patients will experience satisfying oncological outcome. This assumption is supported by a small feasibility study [6], where Richetti et al. analysed VMAT in 45 patients with preoperative CRT of LARC and demonstrated improved dose conformality and a tumor downstaging, comparable to previously published data for conventional RT. Furthermore the favorable oncological outcome in patients treated with IMRT for rectal cancer [9, 19] and other tumors [20–23] supports a potential benefit for VMAT-treated patients. IMRT and VMAT have comparable dosimetric characteristics [17], but IMRT is already used for quite some time and therefore patient data with longer follow up periods are available [24].

In addition, for IMRT, an excellent toxicity profile was achieved for patients with LARC [9, 10, 25], and other tumor entities [20–23, 26]. Comparisons of IMRT with 3DCRT indicate a reduction of acute gastrointestinal toxicity and treatment breaks [10, 25]. Since the issue has not been addressed in larger cohorts, an ongoing clinical trial aims to compare the acute toxicity rates after IMRT and 3DCRT [NCT02151019]. Considerable differences exist between IMRT and VMAT, namely the reduction of treatment delivery time and the number of applied monitor units [6, 27]. Thus, the clinical comparison of VMAT and 3DCRT must be addressed separately. In general, clinical toxicity data for VMAT are scarce. A favorable acute toxicity profile was described for prostate, non-small cell lung, anal canal and endometrial cancer [28–31], and in the feasibility study of Richetti et al. for LARC [6], where high-grade acute organ toxicity occurred in three patients (7 %), only.

The current study demonstrates the potential of VMAT to substantially reduce high-grade acute organ toxicity, showing a ratio of 20 % for 3DCRT versus 5 % for VMAT. Well-known risk factors for the occurrence of high-grade acute organ toxicity in preoperative CRT of LARC are female gender [32], age ≥70 years [33] and low body mass index [34]. In the current study, the VMAT group showed a significantly higher proportion of patients being older than 70 years and having a low body mass index. Remarkably, the VMAT treatment resulted in lower acute toxicity rates despite the preponderance of features for higher risk of toxicity.

With regard to specific organs, differences were found in high-grade skin reaction (7 % vs. 0 %) and in proctitis (12 % vs. 2 %). The reduction of skin reaction might be explained by the fact that VMAT treatment generally leads to an increase in the volume of normal tissue receiving low-dose irradiation and to a decrease in the volume of normal tissue receiving high-dose irradiation [27]. For IMRT in breast cancer treatment, a reduction in acute skin reaction in comparison to 3DCRT was demonstrated [35] where moist desquamation likely occurred in highest skin dose areas [36]. The reduction in proctitis rates was not described previously in the literature. Our group already demonstrated a significant reduction of high dose areas in the PTV with VMAT plans (V_107 %_ = 0.1 %) in comparison to 3DCRT plans (V_107 %_ = 3.5 %) [7]. The diminution of rectal high-dose areas could lead to lower rates of injury to the rectal wall.

In the current study, no differences between 3DCRT treatment and VMAT treatment were observed regarding hematotoxicity. Altogether, six patients (3 %) developed high-grade hematotoxicity. Mell et al. found the irradiated volume of pelvic bone marrow receiving low-dose irradiation to be predictive for hematotoxicity in the CRT of cervical cancer with IMRT. The authors argued that the use of IMRT might be suitable to reduce hematotoxicity in pelvic RT [37]. However, there are no available data comparing hematotoxicity after 3DCRT and VMAT in a similar patient population. Our findings suggest that, despite the probable increase in irradiated volume of bone marrow with VMAT in comparison to 3DCRT, there is no clinically detectable negative effect on hematotoxicity.

To our knowledge, no published data exist on the late toxicity rates after VMAT in preoperative CRT for rectal cancer. We found significantly lower rates of late toxicity for VMAT in comparison to 3DCRT. High-grade late organ toxicity occurred in 22 % of the 3DCRT patients and in 6 % of the VMAT patients. Altogether, high-grade enteritis, urethral stricture or ureteral stenosis occurred in 10 % of the 3DCRT patients, while none of the VMAT patients experienced these complications. In general, the small bowel and bladder complications occur after organ exposure to RT doses of ≥50Gy [38]. Especially for comparably high dose levels, our group demonstrated a remarkable improvement with VMAT. For the small bowel, the V_40Gy_ was 28.4 % with VMAT plans and 41.8 % with 3DCRT plans. For the urinary bladder, the V_40Gy_ was 66.5 % with VMAT plans and 88.4 % with 3DCRT plans [7]. These findings could explain the absence of enteritis, urethral stricture and ureteral stenosis in VMAT patients.

To address the limitations of the current study, though patients were treated in accordance with the respective protocol, the comparison of 3DCRT and VMAT was not a predefined endpoint of the trial. Thus, a potential bias due to covariates cannot be excluded with absolute certainty. Furthermore, due to the fact that VMAT was introduced into clinical practice only a short time ago, the groups appear different in length of follow-up. As an important concern for the late toxicity data, a prolonged observation period is required. However, the latency to the occurrence of late toxicity in preoperative CRT of LARC is less well known. After RT of cervical cancer, high rates of urinary tract and small bowel complications were found during earlier follow-up. The complication rates sharply declined after 2–3 years [39]. In the current study, 28 patients (35 %) in the VMAT group were observed for a period of >2 years. Since none of these patients experienced high-grade enteritis, urethral stricture or ureteral stenosis, the current findings indicate that VMAT reduces late toxicity in preoperative CRT of LARC. Nevertheless, the outstanding strength of the current study is the homogeneous treatment according to the German CAO/ARO/AIO-04 trial. The presented data highlight the benefits of the VMAT irradiation for LARC on a powerful basis.

## Conclusions

In summary, VMAT treatment in preoperative CRT for LARC showed the potential to substantially reduce high-grade organ toxicity, and lower rates of late toxicity were conceivable. Importantly, we could demonstrate that VMAT irradiation did not impair short-term oncological results. We conclude, that the delivery of preoperative RT using VMAT may pave the way for more efficient systemic therapies, and improved patient survival in the multimodal treatment of LARC.

## Additional file

Additional file 1: Table S1. Hematotoxicity. (PDF 41 kb)

## Abbreviations

VMAT: Volumetric modulated arc therapy
CRT: Chemoradiotherapy
LARC: Locally advanced rectal cancer
3DCRT: 3D conformal radiotherapy
TRG: Tumor regression grading
TME: Total mesorectal excision
RT: Radiotherapy
IMRT: Intensity-modulated radiotherapy
CTV: Clinical target volume
PTV: Planning target volume
