# Supplementary material for: Reduced toxicity in the treatment of locally advanced rectal cancer: a comparison of volumetric modulated arc therapy and 3D conformal radiotherapy
Source: BMC Cancer. 2015 Oct 20;15:750. doi: 10.1186/s12885-015-1812-x (PMC4617910; doi:10.1186/s12885-015-1812-x)
Supplement: Additional file 1: Table S1. — Hematotoxicity. (PDF 41 kb) [file 12885_2015_1812_MOESM1_ESM.pdf]

Additional Table 1. Hematotoxicity

Toxicity grade	3D conformal radiotherapy No.	3D conformal radiotherapy %	Volumetric modulated arc therapy No.	Volumetric modulated arc therapy %	Chi-square, p	Kruskal-Wallis, p
Anemia						
≥ 3	1	1	1	1	0.8426	
0	51	48	30	37		0.2417
1	44	41	45	56		
2	11	10	5	6		
3	1	1	1	1		
Leucopenia						
≥ 3	3	3	1	1	0.4603	
0	52	49	31	38		0.3640
1	44	41	39	48		
2	8	8	10	12		
3	2	2	0	0		
4	1	1	1	1		
Thrombopenia						
≥ 3	1	1	0	0	0.3830	
0	89	83	66	82		0.6186
1	17	16	15	19		
4	1	1	0	0		
Any kind of hematologic toxicity*						
≥ 3	4	4	2	3	0.6240	
0	29	27	13	16		0.4010
1	58	54	52	64		
2	16	15	14	17		
3	3	3	1	1		
4	1	1	1	1		

*The highest score of any hematologic toxicity per patient.

You created this PDF from an application that is not licensed to print to novaPDF printer (http://www.novapdf.com)
